# Supplementary material for: SubVis: an interactive R package for exploring the effects of multiple substitution matrices on pairwise sequence alignment
Source: PeerJ. 2017 Jun 27;5:e3492. doi: 10.7717/peerj.3492 (PMC5490468; doi:10.7717/peerj.3492)
Supplement: Supplemental Information 1 [file peerj-05-3492-s001.gz › SubVis/inst/doc/SubVis.html]

# SubVis

#### *Scott Barlowe, Heather Coan, and Robert Youker*

#### *2017-05-08*

About

Description

Other Project Pages

Known Issues

Included Data

Getting Started

Options Tab

VIZ Tab: Overview

VIZ Tab: Detail

VIZ Tab: Search

Error Messages

Notes

References

### About:

```
  SubVis version 2.0.2
  Scott Barlowe, Heather Coan, and Robert Youker
  Western Carolina University
  Uses: R (>= 3.3.0), JavaScript
  Licensing:  GNU General Purpose License
```

### Description:

```
  Software tool for visual analysis of substitution matrix 
  effects on pairwise protein sequence alignment.  Utilizes 
  Shiny for interface components, R for alignment processing, 
  and JavaScript for visualization.
```

### Other Project Pages:

```
  1.  https://github.com/sabarlowe/SubVis
```

### Known Issues:

```
  None
```

### Included Data:

```
  Location:  (extdata/Example_custom_matrix and 
              extdata/Example_FASTA_files)

  1.  Four protein sequences in FASTA format 
        (gpr12.fasta, gpr6.fasta)
        (Human.fasta, Xenla.fasta)
        (fNucl.fasta, pFalc.fasta)
        
  2.  A custom matrix developed by Rios et al. (gpcrtm.txt)
      A scaled and scaled adjusted matrix reported by 
        Yu and Altschul (scaled_BLSM.txt, scaled_adj_BLSM.txt)
      The DISORDER matrix proposed by Radivojac et al. 
        (disorder_mat.txt)
        
  3.  BLOSUM62 matrix used for varying penalties (BLS.txt)
  
  4.  Three custom master files (masterFile_gpcrtm.txt, 
        masterFile_scaled_adj_bls.txt, and masterFile_disorder.txt) 
        listing the file names of custom matrices and their associated 
        gap and extension costs
```

### Getting Started:

```
  (Requires availability of a web browser):

  1.  Install R version >= 3.3.0

  2.  Install required R packages (and any dependencies):

      'shiny' 
    
      'Biostrings'

  3.  Install and load SubVis package

  4.  Launch SubVis at the RStudio prompt with

        '> SubVis::startSubVis()'
      
  NOTE:   ENTERING SEQUENCE TEXT (INSTEAD OF FILE UPLOAD)
          AND USING CUSTOM MATRICES REQUIRES READ/WRITE
          PERMISSIONS IN THE SUBVIS INSTALLATION DIRECTORY
```

### Main Tabs: Options, VIZ, and Help

```
  After launching, there are three tabs:  Options, VIZ, and 
                                          Help
```

### Options Tab: Loading Data, Matrices, and Parameters

```
  The Options tab includes the following features/requirements:
  
  1.  Protein sequences (one per file) must be in FASTA 
        format.  There are three sets of example FASTA files 
        in the extdata/Example_FASTA_files folder.
    
  2.  Sequences can be loaded by either entering the text
        (including by copy/paste) or by file selection.  
        In the case of text entry, text files with the FASTA
        sequences are created in the extdata/Example_FASTA_files 
        directory located in the SubVis installation directory.  
        These files can be saved for future use.
      
  NOTE:   ENTERING SEQUENCE TEXT (INSTEAD OF FILE UPLOAD) 
          REQUIRES READ/WRITE PERMISSIONS IN THE SUBVIS 
          INSTALLATION DIRECTORY
    
  3.  An error message will be generated if
        a.  Either textbox or file selection is empty
        b.  Spaces (other than newlines) are in the sequences
        c.  The header and sequences of both are identical
        d.  The sequences are identical

  4.  BLOSUM and PAM matrices can be selected by checking 
        the appropriate box.  Gaps and extension costs can be
        changed with the associated text boxes.
    
        An error message will be generated if
          a.  The gap entry is empty or is not a number
          b.  The extension entry is empty or is not a number 
           
  5.  Multiple custom matrices in the form of the 
        predefined matrices can be loaded from 
        text files.  The basic form is 
  
        First row --> Characters representing lookup
                        table
        First col --> Transpose of first row starting
                        at the second row
                        
                        A    B   C   D   E   F . . .
        A   
        B
        C
        D         
        E
        F
        ...
        
        All other entries (intersection of amino acids)
                        are substitution values

        Custom matrices are loaded by selecting a master file.
        This file lists the filenames of the custom matrices.  
        Both files should be located in the  
        extdata/Example_custom_matrix folder located in the 
        SubVis installation directory.  The format is:
    
          custom_matrix_name0 gap_cost0 extension_cost0
          custom_matrix_name1 gap_cost1 extension_cost1
          custom_matrix_name2 gap_cost2 extension_cost2
      
                      ...and so on...
    
        Spaces separate the custom matrix name, gap cost, and 
        extension cost for each matrix.  
        
        EACH LINE MUST END WITH A NEWLINE CHARACTER TO AVOID 
        ERRORS/WARNINGS.  THIS INCLUDES THE LAST LINE LISTING 
        A CUSTOM MATRIX.  THERE MUST BE A SINGLE BLANK LINE
        AFTER THE LAST ENTRY.
    
        In the display, the custom matrix is labeled "CM" 
        followed by the number associated with its place in 
        the order matrices are listed in the custom matrix 
        master file (for example, CM0, CM1, etc.).
    
        An error message will be generated if
        a.  The custom matrix radio button is set to "ON" and the 
            file selection is empty
        b.  The format of the custom matrix is not acceptable 
        
  NOTE:   USING CUSTOM MATRICES REQUIRES READ/WRITE
          PERMISSIONS IN THE SUBVIS INSTALLATION DIRECTORY

  6.  The score type can be selected with the drop-down menu
    
  7.  After all parameters are entered, clicking the "GO" button 
        located under the "PERFORM ALIGNMENT" label will change 
        the view to the Overview visualization

  Note: Each time a change in the Options tab is made, 
        the "GO" button must be clicked
```

### VIZ Tab: Overview, Detail View, and Search View

```
  There are three options that can be selected with a drop-down 
    menu:  Overview, Detail view, and Search view
```

### Overview:

```
  Each row (except the last) represents a percent identity 
    type.  Within each row, the PID for the selected matrices 
    are sorted.  The last row is the sorted overall score. 
    
    PID types include the following variances in denominator
      calculations as investigated by May and by Raghava
      and Barton:
    
    PID 1 - Denominator should be defined as aligned positions
              plus internal gap positions.
    PID 2 - Denominator should be defined as aligned positions.
    PID 3 - Denominator should be defined as the length of the shorter     
              sequence.
    PID 4 - Denominator should be defined as the average of the two 
              sequences.

  All PID colors are normalized together.  The score colors 
    are normalized separate from the PIDs.  A legend in the 
    bottom left shows the color scheme along with the minimum 
    and maximum values for the PID and score.
  

  Interaction:  Mouse move

  1.  When the mouse moves over a matrix type, the 
        corresponding matrix in the other rows are identified.  
        At the same time, detail information for the selected 
        matrix (matrix type, percent identity, and score) are 
        shown in the bottom right.  When the mouse moves over
        the overall score, PID 4 for the selected matrix is
        shown.


  Interaction:  Size

  1.  '+' enlarges the displayed items and '-' shrinks the 
        displayed items
```

### Detail View:

```
  The overall score is located under the matrix type for each 
    alignment pair along the left side.  Amino acids are represented 
    by a color-coded box.
  

  Interaction:  Size

  1.  '+' enlarges the displayed items

  2.  '-' shrinks the displayed items


  Interaction:  Mouse over an amino acid

  1.  A histogram displays the number of each amino acid in that 
        column

  2.  The log-odds score (or any substitution value entered 
        into a custom matrix) and the amino acid substitution 
        are displayed under the score for each alignment

  3.  The amino acid, log-odds score (or any substitution value 
        entered into a custom matrix), and the amino acid 
        substitution are shown in the top right
  
  4.  The gap and extension costs for the selected amino acid 
        (under the mouse) are shown in the top left


  Interaction: Classification

  1.  Amino acids can be classified according to [1] and whether 
        an amino acid substitution is conserved (log-odds > 0)
        or not (log-odds < 0)


  Interaction:  Amino Acid Representation
  
  1.  'Alpha On/Off' - Shows amino acid abbreviation instead of boxes


  Interaction: Navigation

  1.  'S' - Go to position 1

  2.  'E' - Go to the end of the alignment with the maximum 
            length

  3.  'U' - Scroll up if all matrix types will not fit onto 
            display space

  4.  'D' - Scroll down if all matrix types will not fit 
            onto display space

  5.  '<' - Go backward in the alignment

  6.  '>' - Go forward in the alignment

  7.  'Pair' - Show both the pattern and subject for each matrix 
               type

  8.  'Patt' - Show only the pattern for each matrix type

  9.  'Subj' - Show only the subject for each matrix type
```

### Search View:

```
  Interaction: POS  (Requires clicking 'GO')

  1.  Go to a position by entering the column number in text 
        box (Requires clicking 'Go')


  Interaction: Search (All require clicking 'Go')

  1.  'NONE' - No search function on (default)

  2.  'INDEL' - Shows the insertions and deletions in red

  3.  'MATCH' - Shows the positions that match in both the 
                pattern and subject

  4.  'SEQ' - The amino acid to be searched for
```

### Error Messages:

```
  Error messages are generated in the following scenarios 
    in the top left of the VIZ tab

  1.  'ERROR: No Sequence Text':
          
          Generated if either sequence text box is empty
  
  2.  'ERROR: No Sequence File':       
          
          Generated if either sequence file selection is 
          empty
          
  3.  'ERROR: Identical Pattern and Subject':  
          
          Generated if the two sequence inputs (header 
          information and sequences) are identical
  
  4.  'ERROR: Identical Sequences':
          
          Generated if the two sequence inputs (sequences 
          only) are the same
  
  5.  'ERROR: Non-Custom Gap':
          
          Bad gap value for the BLOSUM/PAM matrices 
          provided (empty value or not a number)
  
  6.  'ERROR: Non-Custom Ext':            
  
          Bad extension value for the BLOSUM/PAM matrices 
          provided (empty value or not a number)
  
  7.  'ERROR: Bad Sequence or Matrix':         
  
          Error in Biostring's pairwiseAlignment function.  
          Could indicate, among other possibilities, a 
          poorly constructed sequence not indicated by 
          another error message or a poorly constructed 
          custom matrix/custom matrix master file.
  
  8.  'ERROR: No Custom Matrix Selected': 
  
          Custom matrix turned on without selecting a custom 
          matrix master file
          
  9.  'ERROR: Custom Gap':
  
          Bad gap cost for the custom matrix read from the 
          custom matrix file (not a number)
                                      
  10. 'ERROR: Custom Ext':
  
          Bad extension cost for the custom matrix read from the 
          custom matrix file (not a number)
```

### Notes:

```
  1.  Log-odds score (or any substitution value entered 
        into a custom matrix) for pairs involving a gap are
        reported as undefined
```

### References:

```
  1.  Pages, H., Aboyoun, P., Gentleman, R., DebRoy, S.: 
      Biostrings: String Objects Representing Biological 
      Sequences, and Matching Algorithms.  R package version
      2.32.0

  2.  Pearson, W.R., Lipman, D.J.: Improved tools for 
      biological sequence comparison. Proc. Natl. Acad. Sci.
      USA 85(8), 2444{2448 (1988)

  3.  Pommie, C., Levadoux, S., Sabatier, R., Lefranc, G., 
      Lefranc, M.: Imgt standardized criteria for statistical 
      analysis of immunoglobulin v-region amino acid 
      properties. Journal of Molecular Recognition 17(1),
      (2004)

  4.  R Core Team: R: A Language and Environment for 
      Statistical Computing. R Foundation for Statistical 
      Computing, Vienna, Austria (2013). R Foundation for 
      Statistical Computing.  http://www.R-project.org/

  5.  RStudio, Inc.: Shiny: Web Application Framework for R.
      (2014). R package version 0.10.1. 
      http://CRAN.R-project.org/package=shiny
    
  6.  Rios, S., Fernandez, M.F., Caltabiano, G., Campillo,
      M., Pardo, L., Gonzalez, A.: Gpcrtm: An amino acid 
      substitution matrix for the transmembrane region of 
      class a g protein-coupled receptors. BMC Bioinformatics
      16(206). (2015)
      
  7.  Yu, Y., Altschul, S.F.: The construction of amino 
      acid substitution matrices for the comparison of proteins 
      with non-standard compositions.  Bioinformatics 21(7): 
      902-911.  (2004)

  8.  Radivojac, P., Obradovic, Z., Brown, C.J., Dunker, A.K.:
      Improving sequence alignments for intrinsically disordered
      proteins.  Pacific Symposium on Biocomputing 7:589-600.
      (2002)
      
  9.  May, A.: Percent sequence identity; the need to be explicit.   
      Structure, 12(17).  (2004)
      
 10.  Raghava, G. and Barton, G.: Quantification of the variation in 
      percentage identity for protein sequence alignments. BMC 
      Bioinformatics, 7(415).  (2006)
```
